# Decreased Treg Cell and TCR Expansion Are Involved in Long-Lasting Graves’ Disease

**DOI:** 10.3389/fendo.2021.632492

**Published:** 2021-04-12

**Authors:** Ziyi Chen, Yufeng Liu, Shiqian Hu, Meng Zhang, Bingyin Shi, Yue Wang

**Affiliations:** ^1^Department of Endocrinology, The First Affiliated Hospital of Xi’an Jiaotong University, Xi’an, China; ^2^MOE Key Laboratory for Intelligent Networks & Networks Security, School of Electronic and Information Engineering, Xi’an Jiaotong University, Xi’an, China; ^3^Genome Institute, The First Affiliated Hospital of Xi’an Jiaotong University, Xi’an, China; ^4^BioBank, The First Affiliated Hospital of Xi’an Jiaotong University, Xi’an, China; ^5^Department of Endocrinology, Zhejiang Provincial People’s Hospital, Hangzhou, China; ^6^Precision Medicine Center, The First Affiliated Hospital of Xi’an Jiaotong University, Xi’an, China

**Keywords:** Graves’ disease, persistent Graves’ disease, refractory Graves’ disease, regulatory T cell, T cell receptor sequencing, RNA sequencing

## Abstract

Graves’ disease (GD) is a T cell-mediated organ-specific autoimmune disorder. GD patients who have taken anti-thyroid drugs (ATDs) for more than 5 years with positive anti-thyroid stimulating hormone receptor autoantibodies value were defined as persistent GD (pGD). To develop novel immunotherapies for pGD, we investigated the role of T cells in the long-lasting phase of GD. Clinical characteristics were compared between the pGD and newly diagnosed GD (nGD) (N = 20 respectively). Flow cytometric analysis was utilized to determine the proportions of Treg and Th17 cells (pGD, N = 12; nGD, N = 14). T cell receptor sequencing (TCR-seq) and RNA sequencing (RNA-seq) were also performed (pGD, N = 13; nGD, N = 20). Flow cytometric analysis identified lower proportions of Th17 and Treg cells in pGD than in nGD (P = 0.0306 and P = 0.0223). TCR-seq analysis revealed a lower diversity (P = 0.0025) in pGD. Specifically, marked clonal expansion, represented by an increased percentage of top V-J recombination, was observed in pGD patients. Interestingly, pGD patients showed more public T cell clonotypes than nGD patients (2,741 *versus* 966). Meanwhile, RNA-seq analysis revealed upregulation of the inflammation and chemotaxis pathways in pGD. Specifically, the expression of pro-inflammatory and chemotactic genes (*IL1B*, *IL13*, *IL8*, and *CCL4*) was increased in pGD, whereas Th17 and Treg cells associated genes (*RORC*, *CARD9*, *STAT5A*, and *SATB1)* decreased in pGD. Additionally, TCR diversity was negatively correlated with the expression of pro-inflammatory or chemotactic genes (*FASLG*, *IL18R1*, *CCL24*, and *CCL14*). These results indicated that Treg dysregulation and the expansion of pathogenic T cell clones might be involved in the long-lasting phase of GD *via* upregulating chemotaxis or inflammation response. To improve the treatment of pGD patients, ATDs combined therapies, especially those aimed at improving Treg cell frequencies or targeting specific expanded pathogenic TCR clones, are worth exploring in the future.

## Introduction

Graves’ disease (GD) is an organ-specific autoimmune disease that arises due to the breakthrough of tolerance to thyroid stimulating hormone receptor (TSHR). GD has an annual incidence of 20 to 50 cases per 100,000 persons, and females are more susceptible than males (1). This disease is manifested as weight loss, fatigue, heat intolerance, tremor, and palpitations (1). After anti-thyroid drugs (ATDs) withdrawal (12–18 months), 40–50% of GD patients achieve durable remission with euthyroidism, while others will be trapped in long-lasting disease phase (1, 2). Those patients who have been taking ATDs treatment for more than 5 years with positive anti-thyroid stimulating hormone receptor autoantibodies (TRAb) value are classified as persistent GD (pGD) cases, namely refractory GD (rGD) patients in previous studies (3). Although radioactive iodine or thyroidectomy are alternative treatments for pGD patients, either poses a risk of permanent hypothyroidism. Therefore, many pGD patients prefer to continue on ATDs for decades to maintain euthyroidism (4), and this continuous treatment imposes enormous financial and psychological burdens. In addition, based on that GD involved a progressive accumulation of changes in the thyroid glands from short and long clinical course cases (5), there are potential perpetuating factors that maintain the autoimmune response. Considering the unsatisfactory treatment effects of current pGD patients, there is an urgent need to develop novel therapies targeting the underlying accumulated immune factors.

T cells are crucial to the pathogenesis of GD in that they not only infiltrate the thyroid with auto-reactive capacity but also stimulate B cell differentiation toward antibody-secreting plasma cells (6, 7). Once exposed to a specific antigen, naive CD4+ T cells differentiate into different subtypes, including pro-inflammatory T helper 17 (Th17) cells and immunosuppressive regulatory T cells (Tregs) (8). In our previous studies, the presence of decreased Treg proportions and increased Th17/Treg ratios in both animal models and GD patients were confirmed (9, 10), which is consistent with prior achievements (11, 12).

Besides GD, T cell subtypes may also participate in pGD. The -31C/T polymorphism in the *IL1B* gene producing high quantities of IL-1β (a Th17 cell inducer) together with an elevated peripheral percentage of Th17 cells are found (13, 14) in pGD. Additionally, the -3279AA genotype of the Forkhead box protein P3 (Foxp3) gene, which leads to defective transcription of *FOXP3 via* the loss of binding to certain transcription factors (E47 and C-Myb), is present in 11.3% of pGD patients but absent in remittent patients (15). It has been reported that *FOXP3* controls Treg function through co-operation with nuclear factor of activated T cells (*NFAT*), and deficiency of the *FOXP3* gene impairs the suppressor function of Treg cells, which often exacerbates the progression of autoimmune disease (16). Taken together, the results of the above studies indicate that Th17 and Treg cells potentially contribute to the long-lasting phase of GD.

Not only T cell subtypes, but clonal expansion of T cells is also involved in autoimmune diseases (17). T cell receptor (TCR), the diversity of which is a prerequisite for the adaptive immune system to respond to a wide variety of antigens, and the structure of the TCR β gene is a useful tool for identifying clonal expansions of T cells (17, 18). With respect to GD, restricted usage of TCR Vα and TCR Vβ was observed in T cells infiltrating the thyroid together with extrathyroidal sites (19), which emphasizes the crucial role of the TCR repertoire in the pathogenesis of GD. Apart from GD, TCR repertoire is also associated with persistence in other autoimmune diseases. In a study of autologous hematopoietic stem cell transplantation (HSCT) for poor-prognosis multiple sclerosis, non-responders had less TCR diversity than responders early in the course of HSCT (20). In addition, analysis of the CD8+ TCR repertoire in alemtuzumab-treated patients with relapsing-remitting multiple sclerosis (RRMS) who develop a secondary autoimmune disease (vitiligo) revealed greatly increased clonality and reduced repertoire diversity in those patients compared to treatment-naive patients with RRMS (21). Thus, we speculate that TCR repertoire may be involved in the long-lasting phase of GD.

In this study, we investigated the T cell immune mechanisms of the long-lasting phase in GD in terms of the T cell subset frequency (Th17 and Treg cells) and TCR repertoire. The results suggested that dysregulation of Tregs and pathogenic T cell clonal expansion may be involved in the persistence of GD by upregulating chemotaxis or inflammation response. For clinical treatment of pGD patients, precise therapies aimed at improving Treg cell frequencies or targeting specific expanded pathogenic TCR clones are promising approaches for future treatments.

## Materials and Methods

### Study Subjects

We enrolled a total of 20 pGD patients who had taken ATDs for more than 5 years with positive anti-thyroid stimulating hormone receptor autoantibodies (TRAb) value and 34 newly diagnosed GD (nGD) patients who had taken ATDs less than 3 months. These patients were diagnosed according to the guidelines of the American Thyroid Association (ATA). All participants were recruited from the Department of Endocrinology, First Affiliated Hospital of Xi’an Jiaotong University. This study was approved by the Ethic Committee of the First Affiliated Hospital of Xi’an Jiaotong University. We obtained consent from each patient after explaining the purpose of our study. Among these patients, specific clinical characteristics of 20 pGD and 20 nGD patients are presented in **Table 1**. Flow cytometry was performed in 12 pGD and 14 nGD patients. Samples from 13 pGD and 20 nGD patients were subjected to RNA sequencing (RNA-seq) and T cell receptor sequencing (TCR-seq) analysis. The clinical trial registration number is ChiCTR-IPR-16009305.

**Table 1:** Clinical characteristic of enrolled pGD and nGD patients.

Characteristic	Persistent GD (n=20)	Newly diagnosed GD (n=20)	P value
*Basal clinical characteristics*			
Gender (Female/male)	18/2	16/4	0.376^#^
Age (years)	38.3±9.5	40.4±10.8	0.517
Family history of autoimmune thyroid disease (Y/N)	3/17	2/18	0.633^#^
ATD treatment duration (years)	10.28±0.96	NA	NA
Smoking (Y/N)	6/14	9/11	0.327^#^
*Thyroid function*			
TSH (μmol/L)	0.115(0.007-2.480)	0.007(0.007-2.710)	0.067*
FT4 (pmol/L)	21.00(11.00-90.90)	34.60(11.30-200.00)	0.036*
TT4 (pmol/L)	12.35±6.07	20.64±9.06	0.003
TGAb (%)	31.20(3.97-72.70)	16.30(2.24-50.26)	0.134*
TMAb (%)	20.24±12.13	12.24±9.86	0.177
TRAb (U/L)	18.68±2.70	19.53±3.20	0.840
*Other laboratory tests*			
PLT (10^9/L)	239.69±53.38	229.53±43.42	0.459
WBC (10^9/L)	5.46±1.51	6.19±1.38	0.122
LYMPH (10^9/L)	1.77(0.92-2.59)	1.94(0.93-3.14)	0.119*
MONO (10^9/L)	0.31±0.09	0.43±0.17	0.024
NEUT (10^9/L)	3.30±1.25	3.66±1.20	0.427
EO (10^9/L)	0.04(0.00-0.14)	0.07(0.00-0.22)	0.515*
BASO (10^9/L)	0.02(0.00-1.00)	0.02(0.00-0.05)	0.553*
PBMC (10^9/L)	1.86±0.76	2.45±0.64	0.011
CHOL (mmol/L)	3.99±0.59	3.68±1.13	0.733

^#^P value by x^2^-test.

*P value by Mann-Whitney U-test. The rest of P value were by Student’s t-test.

GD, Grave’s disease; TSH, Thyroid stimulating hormone; FT4, Free thyroxine; TT4, Total thyroxine; TGAb, Anti-thyroglobulin auto-antibodies; TMAb, Anti-thyroid microsomal auto-antibodies; TRAb, and anti-thyroid stimulating hormone receptor autoantibodies; PLT, Platelet; WBC, White blood cell; LYMPH, Lymphocyte; MONO, Monocyte; NEUT, Neutrophil; EO, Eosinophil; BASO, Basophil; PBMC, Peripheral blood mononuclear cell; CHOL, Cholesterol. Data were presented as mean±S.D. or median(range).

### Sample Preparation

Nine milliliters of peripheral blood was drawn from each patient and collected in an EDTA anticoagulant tube (BD Biosciences, USA). Next, peripheral blood mononuclear cells (PBMCs) were acquired utilizing Ficoll Paque Plus (GE Healthcare, USA) gradient cell separation according to the manufacturer’s instructions. For RNA-seq and TCR-seq analysis, PBMCs were placed in TRIzol (Ambion, USA), and mRNA was extracted according to the manufacturer’s instructions. Then, the quality and integrity of the total RNA were assessed using an Agilent 2100 Bioanalyzer (Agilent Technologies, USA) and 1.2% agarose gel electrophoresis; the RNA concentration was measured using a NanoDrop 2000 spectrophotometer (NanoDrop Technologies, USA).

### Flow Cytometry

The concentration of isolated PBMCs was adjusted to 10^7^/ml, and 100 μl was used to conduct flow cytometry. Before Th17 cell staining, PBMCs were stimulated with Cell Stimulation Cocktail (Invitrogen, USA) for 6 h at 37°C and 5% CO_2_. Surface staining including CD3, CD8, CD25, and CD4 (all from BD Biosciences, USA) was performed at 4°C for 30 min. After permeabilization with the Transcription Factor Buffer Set (BD Biosciences, USA) according to the manufacturer’s guidelines, anti-Foxp3 and IL17A antibodies (eBiosciences, USA) were incubated at 4°C for 45 min. Four-color flow cytometric analysis was performed using a FACSCalibur flow cytometer (BD Biosciences, USA).

### TCR Sequencing and Analysis

In this study, HTBI primers and Arm-PCR from iRepertoire were used to construct the libraries as previously reported (22). After gel purification, the PCR product was subjected to high-throughput sequencing using the Illumina HiSeq 2000 platform. We amplified all CDR3 sequences present in 33 samples obtained from pGD and nGD patients and then filtered the sequence reads. After the high-quality paired reads were merged using COPE and FqMerger (BGI, China), the results designated as contigs were subsequently aligned to reference TCR Vβ/Dβ/Jβ gene sequences (http://www.imgt.org/download/GENE-DB/) using BLAST. The TCRβ CDR3 regions were identified within the sequencing reads according to the definition established by the International ImMunoGeneTics (IMGT) collaboration. In the end, we identified 62 Vβ and 14 Jβ segments. These results were used to analyze the Vβ and Jβ usage of the CDR3 amino acid clonotypes in each sample. Clones with a frequency of more than 0.1% were considered highly expanded clones (HECs). All TCR-seq raw sequencing data are available through the NCBI SRA accession PRJNA634746.

### RNA Sequencing and Analysis

mRNA was enriched and cleaved into 300-bp fragments using fragmentation buffer. Then, the fragments were used as templates for double-stranded cDNA (dscDNA) synthesis using the SuperScript dscDNA synthesis kit (Invitrogen, USA) according to the manufacturer’s instructions. The dscDNA with indexed adapters was purified and enriched by PCR for 15 cycles and then assessed using a TBS-380 fluorometer, a NanoDrop 2000 spectrophotometer, and an Agilent 2100 Bioanalyzer. The prepared libraries were sequenced using an Illumina HiSeq 3000 sequencer.

For all raw reads, the low-quality bases and adapter sequences were removed, and then the method based on fragments per kilobase of exon per million fragments mapped (FPKM) was used to calculate gene expression. To identify differentially expressed genes (DEGs) between the libraries, we used the DESeq2 package (23). Genes with a fold change ≥2 (|log_2_FC|>1) and a false discovery rate (FDR) <0.05 were considered significant. Two features were extracted from all genes of each group with an unsupervised principal component analysis (PCA) method. Heatmaps and volcano plots were plotted using the pheatmap and ggplot2 packages. We conducted functional enrichment analyses including Gene Ontology (GO) and Kyoto Encyclopedia of Genes and Genomes (KEGG) pathways with the “clusterProfiler” R package (24) and set P value <0.05 as the threshold. Biological processes (BP) and molecular function (MF) were included in the GO enrichment analysis. The R package “GOplot” (25) was used to graphically visualize the results of the GO analyses. The protein-protein interaction (PPI) network was constructed based on data obtained from the SPRING online database (https://string-db.org/) (a confidence score >0.4 was set as the cut-off value) and was displayed using Cytoscape version 3.8.0 (Degree ≥13 regarded as key genes). Pathways with a P-value ≤0.05 were considered significantly enriched. The R package “clusterProfiler” was used to perform GSEA. The gene sets in the Molecular Signatures Database (MSigDB) (26) were selected as the reference gene sets, and P value <0.01 was used as the threshold. All RNA-seq raw sequencing data are available through the NCBI SRA accession PRJNA634746.

### Statistical Analysis

All of the data are presented as the mean, median, or median (range), depending on whether they followed a normal distribution. Statistical analysis was performed using R 3.6.2, SPSS (version 23, IBM, USA) and Prism (version 7, GraphPad, USA). Categorical variables were analyzed by the χ^2^ test. For continuous variables, Student’s t-test (normal distribution determined by Kolmogorov-Smirnov Test) or the Mann-Whitney U-test (non-normal distribution determined by Kolmogorov-Smirnov Test) was adopted. Two-sided P values <0.05 were considered statistically significant. The flow cytometry results were analyzed using FlowJo (version 10, Tree Star, USA). Power analysis was conducted using R 3.6.2 with pwr package 1.3-0 (27) and the results were exhibited in **Table S1**.

## Results

### Clinical Characteristics of pGD and nGD Patients

The clinical characteristics of the patients are shown in **Table 1**. There were 20 pGD patients (age: 38.30 ± 9.510; gender: 2 males and 18 females) and 20 nGD patients (age: 40.40 ± 10.758; gender: 4 males and 16 females). The average time for taking ATD among the pGD patients was 10.29 years. No significant differences were observed in smoking (including passive), thyroid nodules, and thyroid disease family history between the pGD and nGD patients. After more than 5 years of ATDs use, the FT4 and total thyroxine (TT4) levels significantly declined in the pGD patients compared with those observed in the nGD patients (FT4: 21.00 pmol/L *vs* 34.60 pmol/L, P = 0.036; TT4: 12.35 pmol/L *vs* 20.64 pmol/L, P = 0.003), while there were no differences in other thyroid-associated parameters, including TSH, anti-thyroglobulin autoantibodies (TGAb), anti-thyroid microsomal autoantibodies (TMAb), and anti-thyroid stimulating hormone receptor autoantibodies (TRAb). Regarding immune-related cells, while monocyte (MONO) and PBMC counts decreased in the pGD group (MONO: 0.43 × 10^9^/L *vs* 0.31 × 10^9^/L, P = 0.024; PBMC: 2.45 × 10^9^/L *vs* 1.86 × 10^9^/L, P = 0.011), no obvious differences were observed in the quantities of platelets, white blood cells, lymphocytes, basophils, or eosinophils between the groups. Additionally, there was no significant difference in cholesterol concentration between the two groups, which is consistent with our previous finding that high pretreatment cholesterol levels were not correlated with the efficiency of intravenous methylprednisolone in thyroid-associated ophthalmopathy (28).

### Flow Cytometric Analysis Identified Decreased Frequencies of Th17 and Treg Cells in pGD Patients

**Figure 1:**
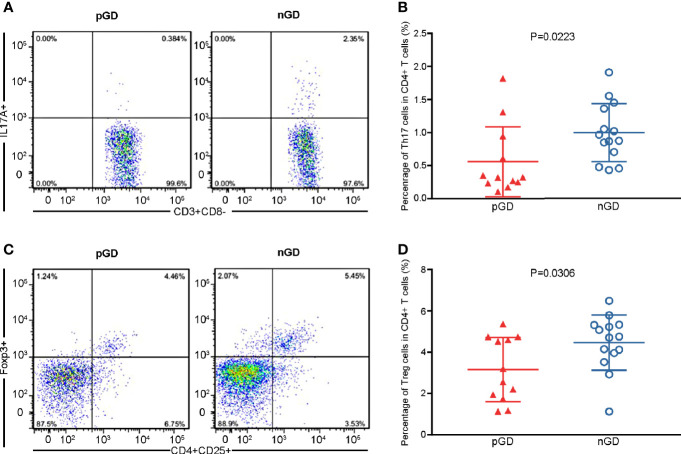
Flow cytometric analysis confirmed declined frequencies of Th17 and Treg cells in pGD patients. **(A, C)** Representative flow cytometry plots of **(A)** Th17 cells (IL17A+) gated in CD3+CD8− T cells and **(C)** Treg cells (CD25+Foxp3+) gated in CD4+ T cells from pGD (left) and nGD (right) patients respectively. The numbers denote the percentage of cells in each rectangle gate. **(B, D)** Whisker plots showed percentage of **(B)** Th17 cells and **(D)** Treg cells in CD4+ T cells in pGD (triangle, N = 12) and nGD (circle, N = 14) patients. Data were exhibited as mean with SD with individual values. pGD, persistent Graves’ disease; nGD, newly diagnosed Graves’ disease; SD, standard deviation.

Considering that Th17 and Treg potentially contribute to the persistence of GD, we conducted flow cytometry of Th17 cells (CD3+CD8−IL17A+, **Figure S1A**) and Treg cells (CD4+CD25+FOXP3+, **Figure S1B**) in 12 pGD and 14 nGD patients. Compared with the nGD group, the proportion of Th17 cells among CD4+ T cells was significantly decreased in the pGD group (0.4595 *vs* 0.9195%, P = 0.0307, **Figures 1A, B**). Moreover, the percentage of Tregs was significantly diminished in the pGD group (3.163 *vs* 4.466%, P = 0.0306, **Figures 1C, D**).

### Restricted TCR Clonotypes Were Revealed in pGD Compared With nGD Patients

**Figure 2:**
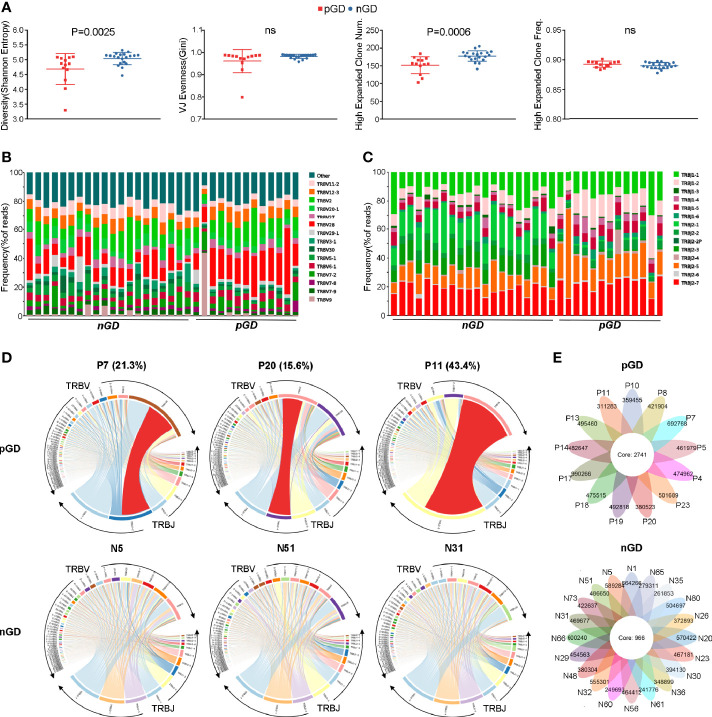
Restricted TCR clonetype were revealed in pGD compared with nGD patients. **(A)** Whisker plots showed Shannon entropy, Gini, high expanded clone number, as well as high expanded clone frequency in pGD patients (red, N = 13) compared with nGD patients (blue, N = 20). Data were exhibited as mean with SD with individual values. **(B, C)** TCR **(B)** V segment and **(C)** J segment frequencies of reads in pGD patients (N = 13) compared to nGD patients (N = 20). Columns represent individual samples. The colors in the bars within each column indicate different V and J segments, and were denoted on the right. **(D)** TRBV and TRBJ gene segment usage and V-J recombination were illustrated by circos plots in sample pGD patients (upper) represented by P7, P20, P11 and nGD patients (bottom) represented by N5, N51, N31. The TRBV and TRBJ genes were clockwise arranged in the order of their frequency from low to high. A VJ recombination was illustrated by colored curved paths whose thickness represents their frequencies in TCR repertoires. **(E)** Flower plots presented the amount of the overlap (core) and individual (petal) in TCR clonotypes in pGD patients (upper, N = 13) and nGD patients (bottom, N = 20), respectively. Freq, frequency; pGD, persistent Graves’ disease; nGD, newly diagnosed Graves’ disease; SD, standard deviation; TCR, T cell receptor.

Given the potential role of TCR repertoire in the long-lasting phase of GD, we decided to perform TCR-seq analysis of PBMCs from pGD and nGD patients (N = 13 and 20, respectively). Compared with the nGD patients, the pGD patients showed decreased Shannon entropy (an index to evaluate TCR diversity), which suggested the persistence of expanded clones in the TCR repertoire of pGD (P = 0.0025, **Figure 2A**). There were no differences in the V-J evenness (Gini coefficient) and frequency of HECs between the two groups. However, the diminished diversity was confirmed by the reduced quantity of HECs in pGD compared to nGD patients (P = 0.0006, **Figure 2A**). Lower diversity of the TCR repertoire may reflect more recognition of certain specific antigen and less responses to diversified antigens. Such restricted recognition implied T cell clones targeting these antigens undergo expansion, which is also called clonal expansion (29). Subsequently, we assessed the gene usage in these two distinct groups. Analysis of the frequency of TRBV and TRBJ segment suggested that TRBV28, TRBJ1-2, and TRBJ2-5 (P = 0.0021, 0.00098, and 0.022, respectively) were preferentially used in pGD patients, while TRBV20-1 and TRBJ2-1 (P = 0.00026 and <0.0001, respectively) were preferred in nGD patients (**Figures 2B, C**; **Figure S2**). In the pGD samples, the top V-J recombination was often observed as a large percentage, such as in pGD patients R11 (43.4%), R7 (21.3%), and R20 (15.6%) (**Figure 2D**). The clonal expansion in pGD patients potentially resulted from persistent exposure to the pathogenic auto-antigen. TCR clones shared by more than one individual are generally considered public. Compared with the nGD patients (N = 966), the sharing of public clones was increased among the pGD patients (N = 2,741), suggesting a selection advantage of public clones (**Figure 2E**). Therefore, compared to the nGD patients, a more skewed TCR repertoire, represented by less TCR diversity, a larger percentage of top V-J combinations, and more public clones, was observed in pGD patients.

### Transcriptional Profiling of PBMCs in pGD and nGD Patients

**Figure 3:**
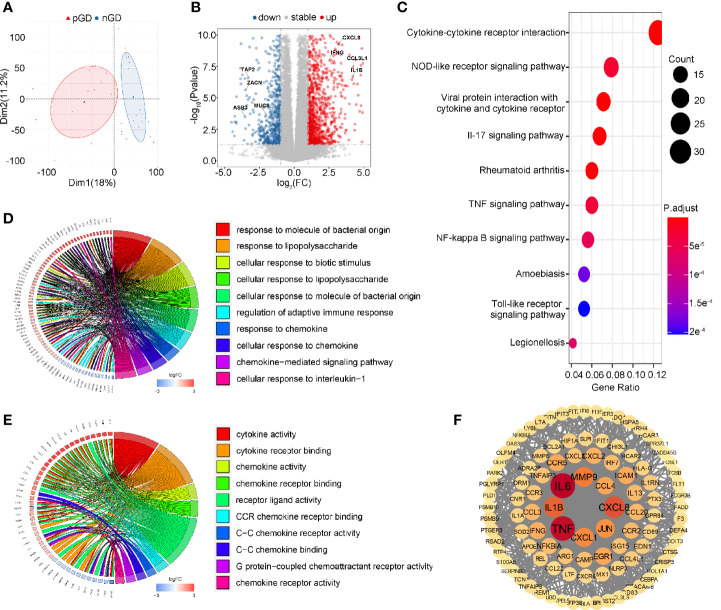
Transcriptional profiling of PBMCs in pGD and nGD patients. **(A)** PCA plot showed obvious differences with all expressed genes in pGD and nGD group. Red triangle represented pGD and blue circle for nGD. **(B)** Volcano plot showed DEGs between pGD and nGD group. Blue dots represented downregulated genes (N = 375) and red dots were upregulated genes (N = 696). The texts denoted in the plot were names for corresponding genes. **(C)** The top 10 most significant KEGG pathways for all DEGs. Size of bubble represented counts of the DEGs in corresponding pathway and color of bubble meant adjust p value. **(D, E)** GO enrichment analysis of DEGs among **(D)** biological processes and **(E)** molecular functions. Circos plots showed the relationship between genes and GO terms. The top 10 most significant GO processes were shown in the plot retrospectively. **(F)** PPI network of potential key DEGs (degree ≧13). Size and color of each gene represented the its degree score. pGD, persistent Graves’ disease; nGD, newly diagnosed Graves’ disease; PCA, principal component analysis; DEGs, differentially expressed genes; KEGG, Kyoto Encyclopedia of Genes and Genomes; GO, Gene ontology; PPI, Protein-protein interaction.

To further explore the potential function of decreased Treg cell frequency and marked TCR clonal expansion in pGD, we performed transcriptional profiling of PBMCs from 13 pGD and 20 nGD patients. The principal component analysis (PCA) score trajectory plot of the persistent group did not overlap with that of the newly diagnosed group, indicating that there were significant differences in the transcriptional profiles between the two groups (**Figure 3A**). Combined with the hierarchical cluster analysis of the global gene expression profiles, these results revealed the existence of discrimination between pGD and nGD patients (**Figure S3**).

Compared to the nGD patients, 1,071 differentially expressed genes (DEGs) were identified in the persistent group, including 375 downregulated genes and 696 upregulated genes (**Figure 3B**). For the Kyoto Encyclopedia of Genes and Genomes (KEGG) pathways, cytokine-cytokine receptor interaction was most often enriched by DEGs (**Figure 3C**). Gene Ontology (GO) analysis of the DEGs revealed that regulation of adaptive immune response, immune activities associated with chemokines and pro-inflammatory IL-1β were enriched among biological processes (BP) (**Figure 3D**). Likewise, cytokine and chemokine related activities were the most significant among molecular functions (MF) in the GO analysis (**Figure 3E**). A protein-protein interaction (PPI) network of DEGs was constructed, and 103 genes were identified as potential hub genes (Degree ≧13), where those genes with highest degree were inflammation and chemotaxis related including *IL6*, *TNF*, *CXCL8*, and *IL-1β* (**Figure 3F**). Taken together, these results suggested that inflammation and chemotaxis are correlated with the long-lasting phase of GD.

### Specific Gene Expression in pGD Patients

**Figure 4:**
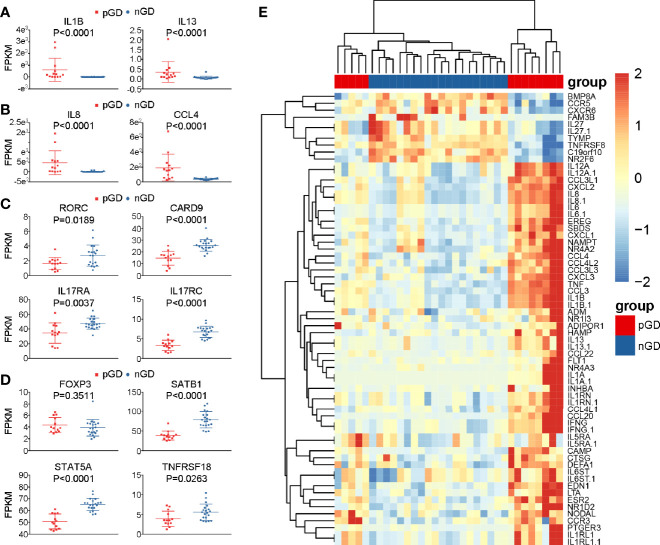
Specific genes expression in pGD compared with nGD patients. **(A–D)** Whisker plots indicated normalized gene expression associated with **(A)** inflammation **(B)** chemotaxis **(C)** Th17 cells **(D)** Treg cells between pGD (red, N = 13) and nGD (blue, N = 20) samples. Data were exhibited as mean with SD with individual values. **(E)** The hierarchical clustering of 13 pGD and 20 nGD patients by differentially expressed immune related genes. The heatmap showed, for each gene, the scaled expression in each patient. pGD, persistent Graves’ disease; nGD, newly diagnosed Graves’ disease; SD, standard deviation; FPKM, fragments per kilobase of exon per million fragments mapped.

Subsequently, we performed a detailed analysis of specific gene expression in the persistent and newly diagnosed group. Compared with the nGD patients, pro-inflammatory genes, including *IL1B* and *IL13*, were upregulated in the persistent group (*IL1B*: P < 0.0001; *IL13*: P < 0.0001, **Figure 4A**), which was consistent with previous reports (12, 30). In addition, upregulation of chemokines (*IL8* and *CCL4*) was observed in pGD patients (*IL8*: P < 0.0001; *CCL4*: P < 0.0001, **Figure 4B**). These results were in agreement with a previous report that *IL8* and *CCL4* are associated with relapse in autoimmune polychondritis (31).

Apart from the increased expression of pro-inflammatory and chemokine genes, consistent with our flow cytometric results, the expression of Th17- and Treg-related genes was decreased in pGD compared with that observed nGD patients. Genes associated with Th17 cells, such as *RORC* (P = 0.0189), *CARD9* (P < 0.0001), *IL17RA* (P = 0.0037), and *IL17RC* (P < 0.0001), were significantly downregulated in the persistent group (**Figure 4C**). In addition, although the master regulator gene *FOXP3* in pGD patients presented no difference compared to the newly diagnosed group, the expression levels of genes that are crucial in Treg differentiation and maintenance, including *SATB1* (P < 0.0001), *STAT5A* (P < 0.0001), and *TNFRSF18* (P = 0.0263), were obviously decreased (**Figure 4D**). In addition to the specific genes observed above, samples from pGD patients expressed higher levels of chemotaxis and inflammation molecules, including *TNF*, *IL-6*, *CXCL2*, and *CXCL3* (**Figure 4E**).

### Robust Inflammatory and Cytokine Responses Pathways Are Prominent in pGD Patients With Lower FOXP3 and IKZF2 Expression

**Figure 5:**
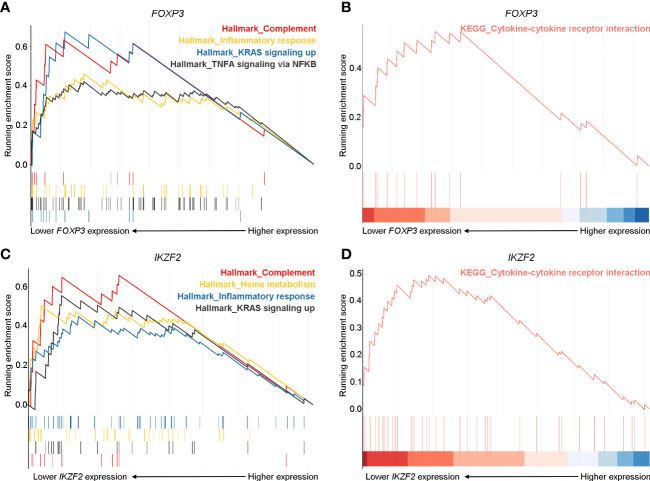
Robust inflammatory and cytokine responses pathways were prominent in pGD patients with lower *FOXP3* and *IKZF2* expression. **(A, C)** Gene set enrichment analysis (GSEA) showed complement, inflammatory response, KRAS signaling up pathways in hallmark gene set were most enriched in pGD patients with **(A)** lower *FOXP3* and **(C)** lower *IKZF2* expression. **(B, D)** GSEA showed cytokine-cytokine receptor interaction pathway in Kyoto Encyclopedia of Genes and Genomes (KEGG) pathways was obviously enriched in pGD patients with **(B)** lower *FOXP3* and **(D)** lower *IKZF2* expression. pGD, persistent Graves’ disease.

To further validate the crucial role of Treg cells in the pathogenesis of pGD and elucidate potentially associated functional pathways, we performed GSEA analyses to map the biological processes (**Figure 5**). *FOXP3* and *IKZF2* were previously reported as master genes for the differential and suppressive function of Treg cells and its subset naturally occurring Treg cells (nTregs) (32). Therefore, we divided the pGD patients into two groups based on the expression of these two genes to perform subsequent analyses. In pGD patients with lower *FOXP3* or *IKZF2* expression, inflammatory response, complement, TNFA signaling *via* NFKB in the hallmark gene set together with the cytokine-cytokine receptor interaction pathway in KEGG pathways were significantly enriched (**Figures 5A–D**). However, there was no obvious enrichment in pGD patients with lower *RORC* or *CARD9* gene expression (key genes for Th17 cells) compared with those with higher expression. Taken together, the GSEA results suggested that Treg cells contribute to the long-lasting phase of GD by aggravating the inflammatory response.

### Lower TCR Diversity Is Correlated With Inflammation and Chemotaxis in pGD Patients

**Figure 6:**
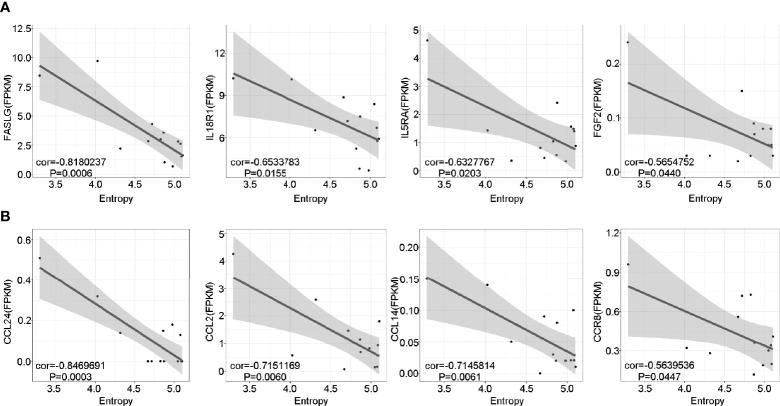
Lower TCR diversity was correlated with inflammation and chemotaxis in pGD. **(A, B)** The correlations of TCR Entropy with the expression of **(A)** inflammation **(B)** chemotaxis associated genes in pGD patients (N = 13) by linear regression analysis using Pearson method. The gray zone represented 95% confidence intervals. Texts denoted in the left bottom corner were the correlation coefficient (cor) and P value. pGD, persistent Graves’ disease; TCR, T cell receptor.

To further investigate the potential role of the marked expansion of T cell clones in the long-lasting phase of GD, we conducted linear regression analysis between TCR diversity (as reflected by entropy) and specific gene expression among the pGD patients. The results revealed that TCR diversity exhibited a negative correlation with inflammation-associated genes, including *FASLG* (P = 0.0006), *IL18R1* (P = 0.0155), *IL5RA* (P = 0.0203), and *FGF2* (P = 0.0440) (**Figure 6A**). In addition, chemotaxis-related genes, including *CCL24*, *CCL14*, *CCL2*, and *CCR8*, were significantly negatively correlated with TCR diversity (P = 0.0003, 0.0061, 0.0060, and 0.0447, respectively; **Figure 6B**). In summary, lower TCR diversity was obviously associated with more robust inflammation and chemotaxis among pGD patients.

## Discussion

In the present study we investigated the underlying perpetuating factors that maintain the autoimmune response in GD through flow cytometric, TCR-seq, and RNA-seq analyses, and our findings emphasized the essential role of T cells. To our knowledge, this is the first research that exploring the mechanism of pGD by TCR-seq and RNA-seq. We identified that decreased Treg frequency and a marked expansion of TCR clonotypes were correlated with the long-lasting phase of GD *via* potential aggravation of inflammation and chemotaxis, which shed new light on the treatment for pGD patients.

RNA-seq analysis of PBMCs revealed robust inflammation and chemotaxis in the pGD group compared with that observed in the nGD group. In agreement with our results, the ratio of intracellular TLR7 (iTLR7) and iTLR9 intensities in B cells, which can indicate an excessive release of pro-inflammatory cytokines, were enhanced in pGD patients (33). In addition, serum-derived exosomes from pGD patients stimulated the mRNA expression of pro-inflammatory IL-1β and tumor necrosis factor α (TNF-α) in PBMCs (34). In another study, the *MIG* rs2276886 AG genotype, which regulates the migration of Th17 cells by altering the affinity for C-X-C motif chemokine receptor 3 (CXCR3), was reduced in pGD patients (35). Mechanistically, robust inflammation in the thyroid can be boosted by immune cell infiltration resulting from overexpressed chemotactic molecules. Subsequently, such inflammation will aggravate the autoimmune reaction by amplifying apoptotic signals, cell activation, and tissue remodeling, and may result in the long-lasting phase of GD (36). Taken together, these findings suggest that intensive inflammation and chemotaxis are involved in the progressive evolution of GD.

Given that the primary effector molecular of Th17 cells, IL-17, is associated with inflammation and chemoattraction, we speculate that the robust inflammation and chemotaxis in pGD was mediated by Th17 cells (37). However, the decreases in Th17 cell frequency and related gene expression together with no obvious enrichment in the GSEA results of pGD patients with lower *RORC* or *CARD9* refute our hypothesis. Consistent with our findings, the frequency of the suppressor of cytokine signaling 3 (*SOCS3*) rs4969170 AA genotype, which negatively regulates Th17 generation, was elevated in patients with pGD (38). Previous studies have shown an increase in Th17 cell-related factors in pGD patients compared with remittent patients (12, 13, 39). A possible explanation for this observation is that nGD patients, possessing higher Th17 cells frequencies and levels of the related cytokines CXCL10 and IL23 (40, 41), rather than those remittent ones, were used as controls in the present study, which further resulted in a decreased Th17 ratio after comparison. In that way, the divergence between our results and previous findings can be partially explained.

Furthermore, we identified that descending Treg-related genes and frequencies dominated in pGD patients through RNA-seq and flow cytometric analysis, which further deteriorate the inflammatory and chemotactic state as suggested by both the GSEA results and previous studies (42). Although achievements in Treg alteration for pGD patients are insufficient, studies concerning immune suppression regulation in pGD agree with our findings. The *MIR125A* rs12976445 CC genotype, which is correlated with reduced expression of miR-125a (a negative regulator of activated T cells), together with hsa-miR-98-5p (a negative regulator of immunosuppressive IL-10), were increased in pGD patients compared to that observed in remitted patients (43, 44). Interestingly, under inflammatory environments, loss of Foxp3 expression in Treg cells has been observed, which suggests that impaired Treg cells in pGD can be intrinsic and/or extrinsic (45). Combined with the potential role of inflammation and chemotaxis in the pathogenesis of pGD, the dysregulation of Treg cells is closely associated with the long-lasting phase of GD.

Another assumption of the origin of the excessive inflammatory and chemotactic response is the expansion of unique pathogenic T cell clones in pGD. Indeed, TCR-seq analysis revealed marked expansion of T cell clones and increased public clones in pGD relative to nGD patients. These public T cell clones detected in pGD patients are potentially TSHR-specific and pathogenic similar to experimental autoimmune encephalomyelitis (EAE)-specific T cells with a shared Vβ8.2 segment (46). Mechanistically, antigen-specific T cells can initiate a cascade of immune cell immigration into the antigen-loaded site and further induce inflammation mediated by IFN-γ and TNF-α (47). It is reasonable to infer that the persistent expansion of antigen specific T cell clones in pGD patients will result in accumulated inflammation, chemotaxis, and a refractory autoimmune condition. Such inference can be partially demonstrated by our findings in that the diversity index was positively correlated with the expression of specific pro-inflammatory and chemotactic genes. Collectively, marked clonal expansion of pathogenic T cell clones leads to the long-lasting phase of GD by eliciting more intensive inflammatory and chemotactic responses.

The current study has some limitations. First, the quantity of samples was insufficient, which may have caused our results to be inaccurate to some extent. It is expected that a larger number of samples could be enrolled to perform a more detailed analysis. Second, considering that thyroid is the primary lesion while inaccessible, PBMCs utilized in this research can only partially disclose the root cause of pGD. Third, given the inherent defects of RNA-seq analysis, some alterations that are prominent in certain cell subtypes and frequently repeated in other types may be ignored. Methods such as single-cell RNA-seq could eliminate such weaknesses, and the application of such methods to explore the long-lasting phase of GD is a promising approach.

In summary, marked expansion of pathogenic T cell clones in concert with dysregulated Treg cells contribute to the long-lasting phase of GD by presumably eliciting excessive inflammation and chemotaxis responses. For patients with pGD, precise therapies aimed at improving Treg cell frequencies or targeting specific expanded pathogenic TCR clones are promising approaches for future treatments.

## Data Availability Statement

The datasets presented in this study can be found in online repositories. The names of the repository/repositories and accession number(s) can be found below: NCBI and PRJNA634746 https://www.ncbi.nlm.nih.gov/bioproject/PRJNA634746.

## Ethics Statement

The studies involving human participants were reviewed and approved by Ethic Committee of the First Affiliated Hospital of Xi’an Jiaotong University. The patients/participants provided their written informed consent to participate in this study.

## Author Contributions

ZC, YW, and BS conceived and designed the study. BS, SH, and ZC screened participants for study entry. ZC, YL, and YW processed the data. SH, MZ, and ZC collected samples and data. ZC and SH performed FACS analysis. ZC, YW, YL, and BS wrote the manuscript. All authors contributed to the article and approved the submitted version.

## Funding

This work was supported by the National Key R&D Program of China [grant NO. 2018YFC1311500 (BS)], National Science Foundation of China (NSFC) [grant NO. 81970679 (BS), 81500690 (YW)], Natural Science Foundation of Shaanxi Province [2018JM70990 (YW)], the Fundamental Research Funds for the Central Universities [1191329875 (YW)], China Postdoctoral Science Foundation [224646 (YW)], and Clinical research award of the first affiliated hospital of Xi’an Jiaotong University (XJTU1AF-CRF-2019-020).

## Conflict of Interest

The authors declare that the research was conducted in the absence of any commercial or financial relationships that could be construed as a potential conflict of interest.

## Supplementary Material

Supplementary Figure 1 Flow cytometry analysis of Th17 and Treg cells. **(A, B)** Gating of **(A)** Th17 cells and **(B)** Treg cells.

Supplementary Figure 2 Global gene expression profiles from pGD and nGD samples. Pairwise Pearson correlations of expression of genes with top 500 mad (median absolute deviation) value between individual patient samples. Samples were organized by hierarchical clustering based on their Pearson coefficients across samples. The disease is indicated by red (pGD, N = 13) and blue (nGD, N = 20) squares on the top and left of the matrix. The patient number for each sample is indicated to the bottom of the matrix. pGD, persistent Graves’ disease; nGD, newly diagnosed Graves’ disease.

Supplementary Figure 3 Specific TRBV and TRBJ segment usage in pGD and nGD samples. **(A, B)** Boxplots showed the frequency of **(A)** TRBV28, TRBJ1-2, TRBJ2-5, **(B)** TRBV20-1, TRBJ2-1 in all reads between pGD patients (red, N = 13) and nGD patients (blue, N = 20). Boxplot with plot center and box corresponding to median and extremum respectively and include individual data points. pGD, persistent Graves’ disease; nGD, newly diagnosed Graves’ disease.
