# Supplementary figures and images for: Decreased Treg Cell and TCR Expansion Are Involved in Long-Lasting Graves’ Disease
Source: Front Endocrinol (Lausanne). 2021 Apr 12;12:632492. doi: 10.3389/fendo.2021.632492 (PMC8074859; doi:10.3389/fendo.2021.632492)

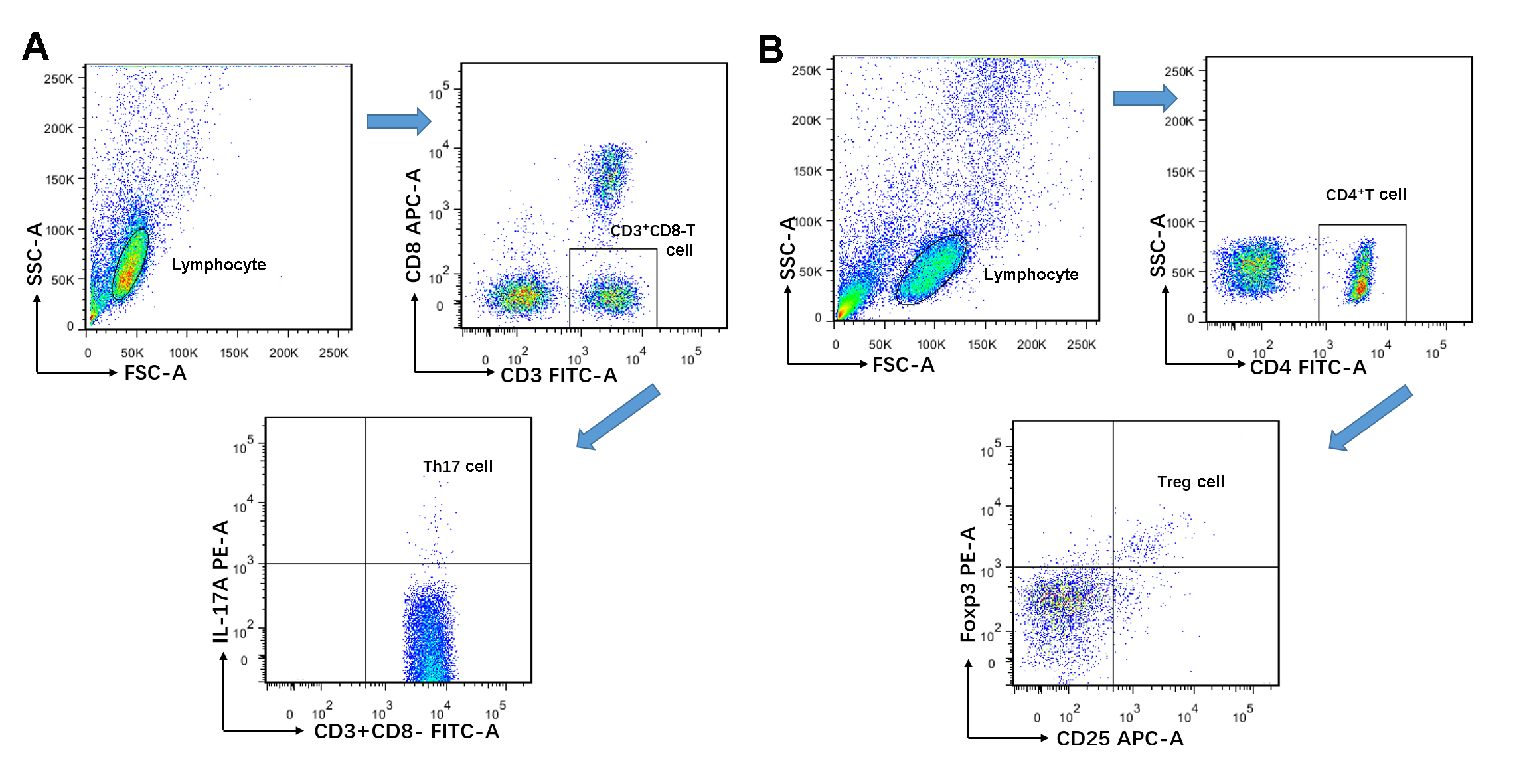

Supplement: Supplementary Figure 1 — Flow cytometry analysis of Th17 and Treg cells. (A, B) Gating of (A) Th17 cells and (B) Treg cells. [file Image_1.tif]

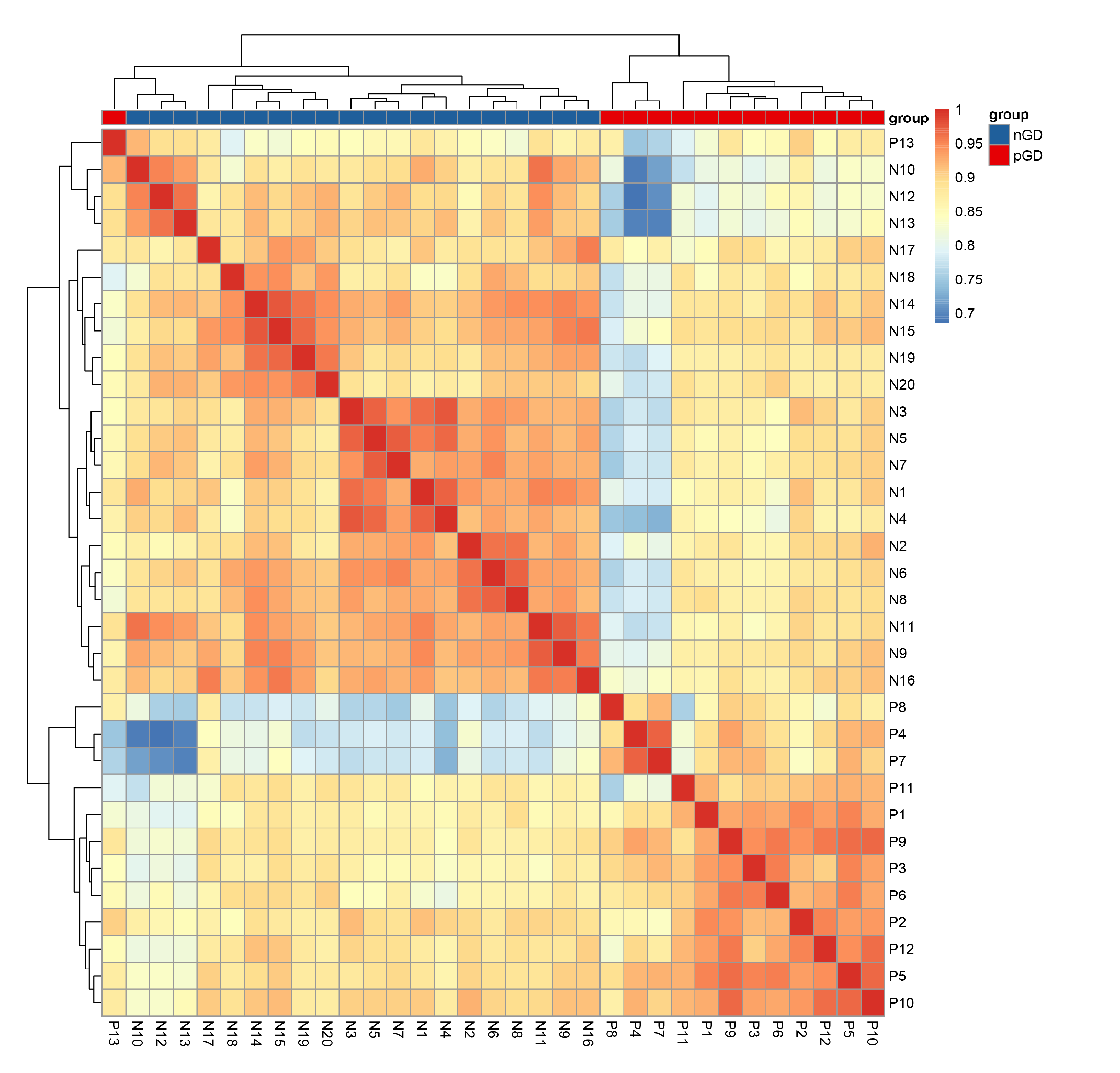

Supplement: Supplementary Figure 2 — Global gene expression profiles from pGD and nGD samples. Pairwise Pearson correlations of expression of genes with top 500 mad (median absolute deviation) value between individual patient samples. Samples were organized by hierarchical clustering based on their Pearson coefficients across samples. The disease is indicated by red (pGD, N = 13) and blue (nGD, N = 20) squares on the top and left of the matrix. The patient number for each sample is indicated to the bottom of the matrix. pGD, persistent Graves’ disease; nGD, newly diagnosed Graves’ disease. [file Image_2.tif]

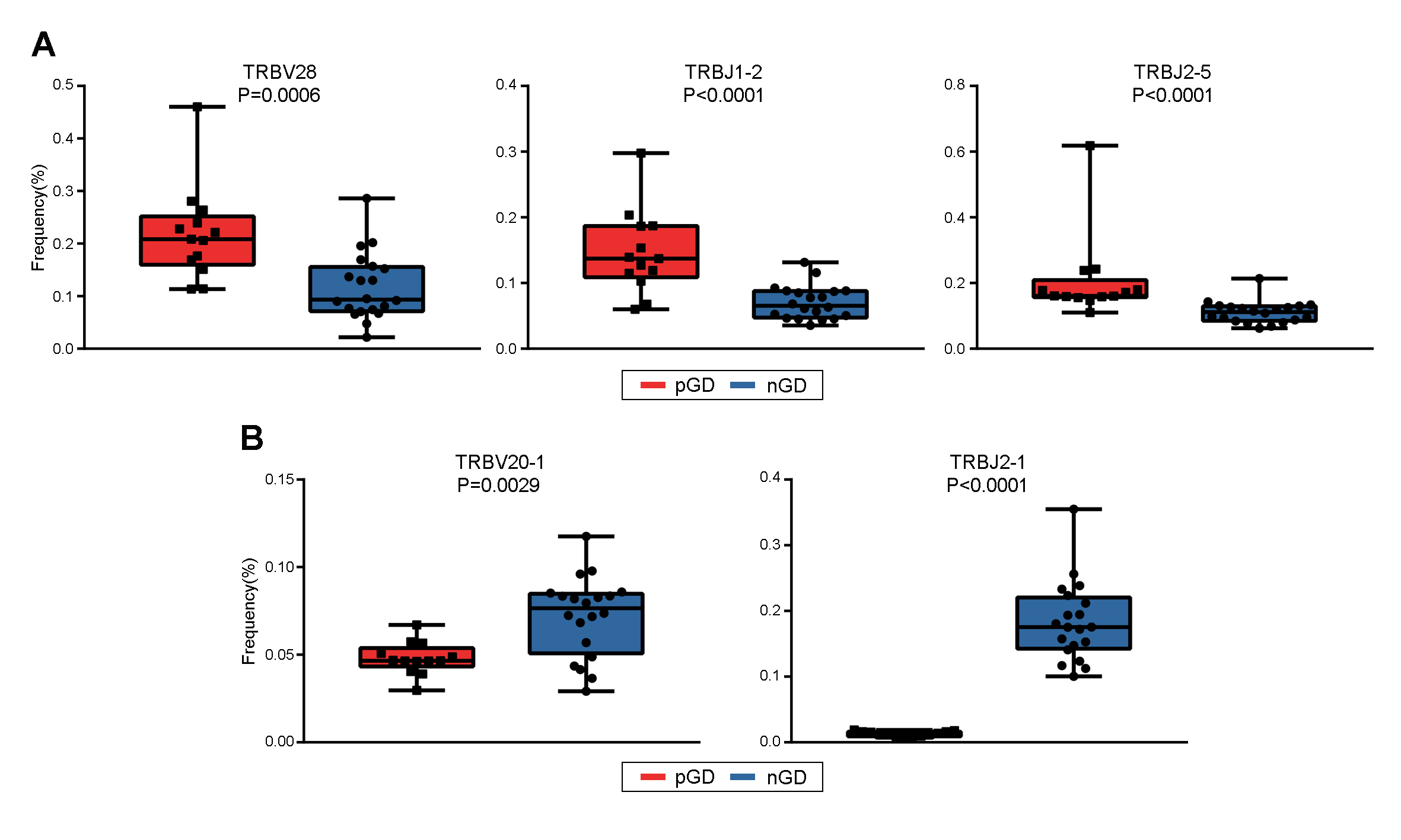

Supplement: Supplementary Figure 3 — Specific TRBV and TRBJ segment usage in pGD and nGD samples. (A, B) Boxplots showed the frequency of (A) TRBV28, TRBJ1-2, TRBJ2-5, (B) TRBV20-1, TRBJ2-1 in all reads between pGD patients (red, N = 13) and nGD patients (blue, N = 20). Boxplot with plot center and box corresponding to median and extremum respectively and include individual data points. pGD, persistent Graves’ disease; nGD, newly diagnosed Graves’ disease. [file Image_3.tif]
