# Supplementary material for: Decreased Treg Cell and TCR Expansion Are Involved in Long-Lasting Graves’ Disease
Source: Front Endocrinol (Lausanne). 2021 Apr 12;12:632492. doi: 10.3389/fendo.2021.632492 (PMC8074859; doi:10.3389/fendo.2021.632492)
Supplement: Supplementary file 4 [file Table_1.docx]

Table S1. Power analysis of enrolled pGD and nGD patients.

| Parameters | Persistent GD | Newly diagnosed GD | Power value |
| --- | --- | --- | --- |
| Age(years) | 38.3±9.5 | 40.4±10.8 | 0.098 |
| TSH(μmol/L) | 0.115(0.007-2.480) | 0.007(0.007-2.710) | 0.301 |
| FT4(pmol/L) | 21.00(11.00-90.90) | 34.60(11.30-200.00) | 0.676 |
| TT4(pmol/L) | 12.35±6.07 | 20.64±9.06 | 0.912 |
| TGAb(%) | 31.20(3.97-72.70) | 16.30(2.24-50.26) | 0.707 |
| TMAb(%) | 20.24±12.13 | 12.24±9.86 | 0.606 |
| TRAb(U/L) | 18.68±2.70 | 19.53±3.20 | 0.055 |
| PLT(10^9/L) | 239.69±53.38 | 229.53±43.42 | 0.099 |
| WBC(10^9/L) | 5.46±1.51 | 6.19±1.38 | 0.344 |
| LYMPH(10^9/L) | 1.77(0.92-2.59) | 1.94(0.93-3.14) | 0.258 |
| MONO(10^9/L) | 0.31±0.09 | 0.43±0.17 | 0.801 |
| NEUT(10^9/L) | 3.30±1.25 | 3.66±1.20 | 0.146 |
| EO(10^9/L) | 0.04(0.00-0.14) | 0.07(0.00-0.22) | 0.339 |
| BASO(10^9/L) | 0.02(0.00-1.00) | 0.02(0.00-0.05) | 0.195 |
| PBMC(10^9/L) | 1.86±0.76 | 2.45±0.64 | 0.693 |
| CHOL(mmol/L) | 3.99±0.59 | 3.68±1.13 | 0.184 |
| Th17 cell/CD4+T cell(%) | 0.4595%±0.1532% | 0.9195%±0.1173 | 0.590 |
| Treg cell/CD4+T cell(%) | 3.163%±0.4487% | 4.466%±0.3579% | 0.592 |

GD: Grave’s disease; TSH: Thyroid stimulating hormone; FT4: Free thyroxine; TT4:Total thyroxine; TGAb: Anti-thyroglobulin auto-antibodies; TMAb: Anti-thyroid microsomal auto-antibodies; TRAb: and anti-thyroid stimulating hormone receptor autoantibodies; PLT: Platelet; WBC: White blood cell; LYMPH: Lymphocyte; MONO: Monocyte; NEUT: Neutrophil; EO: Eosinophil; BASO: Basophil; PBMC: Peripheral blood mononuclear cell; CHOL: Cholesterol. Data were presented as mean±S.D. or median(range).
